# *Lactobacillus plantarum* alleviates high-fat diet-induced obesity by altering the structure of mice intestinal microbial communities and serum metabolic profiles

**DOI:** 10.3389/fmicb.2024.1425764

**Published:** 2024-08-30

**Authors:** Junwen Zhu, Xueying Liu, Naiyuan Liu, Ruochi Zhao, Shuangshuang Wang

**Affiliations:** ^1^Department of Cardiology, The First People’s Hospital of Wenling, Wenling Hospital of Wenzhou Medical University, Wenling, China; ^2^Hunan Provincial Engineering Research Center of Applied Microbial Resources Development for Livestock and Poultry, College of Bioscience and Biotechnology, Hunan Agricultural University, Changsha, China; ^3^Key Laboratory of Precision Medicine for Atherosclerotic Diseases of Zhejiang Province, Affiliated First Hospital of Ningbo University, Ningbo, China

**Keywords:** obesity, probiotics, *Lactobacillus plantarum*, intestinal microbes, fat metabolism, differential metabolites

## Abstract

Obesity, which is always accompanied by disorders of lipid metabolism and dysbiosis of the gut microbiota, has become a global epidemic recognised by the World Health Organisation, necessitating innovative strategies and a globally accepted agreement on treating obesity and its related complications. Probiotics, as major active ingredients in many foods, offer potential as biological treatments for obesity prevention and management. *Lactobacillus plantarum* (*L. plantarum*) possesses a wide range of biological activities and is widely used to alleviate and ameliorate various diseases. This research demonstrated that *Lactobacillus plantarum* reduces the weight increase and fat build-up caused by a high-fat diet (HFD) in mice, while also improving glucose tolerance and insulin sensitivity in obese mice. Results indicated that *L. plantarum* effectively controlled the intestinal microbial community’s structure, counteracted disruptions in gut flora caused by HFD, normalized the Firmicutes to Bacteroidota ratio (F/B), and decreased the prevalence of detrimental bacteria *Desulfovibrio* and Clostridia. Serum metabolomics findings indicate notable alterations in serum metabolites across various groups, notably the increased levels of Isoprothiolane and Inosine, key regulators of lipid metabolism disorders and enhancers of fat burning. These differential metabolites were mainly enriched in unsaturated fatty acid biosynthesis, sulfur metabolism, fatty acid biosynthesis, and purine metabolism. Consequently, we propose that *L. plantarum* has the potential to alter the gut microbial community’s composition, positioning it as a promising option for obesity therapy.

## 1 Introduction

Obesity is defined as excess adiposity and adipose tissue expansion, which occurs through adipocyte hypertrophy and hyperplasia (Cai et al., 2022; Hotamisligil, 2017). Numerous research works suggest that obesity may lead to the emergence of several illnesses (Hoyt et al., 2014), such as metabolic syndrome, Type 2 diabetes mellitus (DMTII) (Youn et al., 2021), heart-related diseases, and fatty liver disease (Hotamisligil, 2017). Statistical data indicates that obesity is responsible for 44% of Type 2 diabetes, 23% of ischaemic heart disease, and as much as 41% of specific cancer types (Boccellino and D'Angelo, 2020). The intestinal microbiota is involved in lipid synthesis and metabolism and is an important component of host energy intake and metabolism (Backhed et al., 2004). It has been demonstrated that the ratio (F/B) of the colonic contents of HFD-induced obese mice (Torres-Fuentes et al., 2017), represented by the Firmicutes and Bacteroidota (Shen et al., 2018), showed a significant increase, which led to metabolic dysregulation and fat accumulation.

With the deeper understanding of a large number of studies, it has been found that probiotics can be used as an effective strategy for obesity prevention (Abenavoli et al., 2019). Probiotics play an irreplaceable role in improving the structure of gut microbial community (Backhed et al., 2004). Studies have proposed that the antagonistic effect that probiotics can have on the growth of pathogenic microorganisms, as well as their ability to modulate the gastrointestinal immune system, results in the modulation of the composition of the gut microbiota and host lipid metabolism (Lee et al., 2018). As a probiotic, *Lactobacillus plantarum* serves as a key component in numerous food items, exhibiting diverse functional characteristics including reducing cholesterol, acting as an antioxidant, and modulating immune responses (Han et al., 2020). It is also able to interact directly with host immune cells and improve intestinal barrier function, which has led to its use in the alleviation and treatment of many diseases, including obesity. A multitude of research indicate s that *L. plantarum* can preserve mitochondrial health and integrity by activating PPAR-α (Cai et al., 2020), simultaneously inhibiting genes related to fatty acid production and the repair of the intestinal barrier. Moreover, *in vitro* and *in vivo* studies have found that *Lactobacillus plantarum* is highly resistant to artificial gastric and intestinal fluids, ensuring that it can survive in large quantities after digestion in the stomach and intestines (Zhang et al., 2022; Rahayu et al., 2021).

This research aimed to explore the capabilities of *L. plantarum*, known for its various bioactivities affecting gut microbiota, in mitigating and treating obesity (Zhou et al., 2021). *L. plantarum* was administered by gavage to HFD-induced obese mice, and the therapeutic effect of *L. plantarum* was determined by recording changes in body weight and detecting subcutaneous and perirenal fat content (Long et al., 2020). Furthermore, alterations in gut flora and metabolic patterns in living organisms were examined through extensive sequencing of colon contents and serum metabolomic studies. This serves as a theoretical foundation for considering *L. plantarum* as a potential treatment option for obesity.

## 2 Materials and methods

### 2.1 Animals

**Figure 1:**
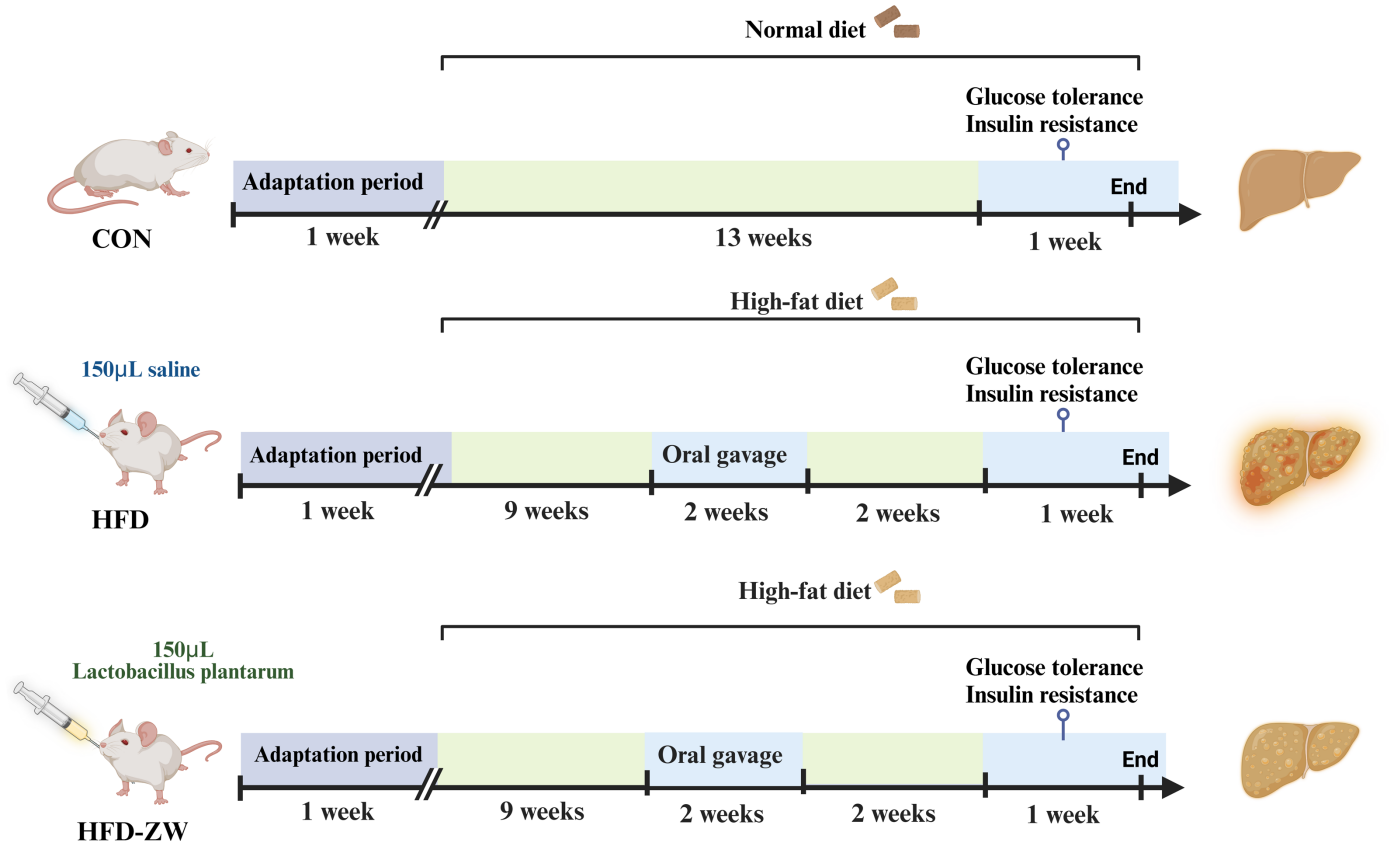
Experimental group assignment and workflow. The 30 mice were divided into three different groups (CON group, HFD group and HFD-ZW group) for 15 weeks.

The animal study adhered to Hunan Agricultural University’s established protocols for laboratory animal care and usage in animal research. All animal experiments were approved by the Animal Ethics Committee of Hunan Agricultural University (approval number: 2023-251). We acquired 30 female ICR mice, each approximately 8 weeks old, from Hunan Sileike Jingda Co (Changsha, China). All animals passed safety quarantine. The mice were kept at a temperature of 25 ± 1°C and on a light or dark cycle for 12 h. The acclimatisation period was 1 week, during which all mice drank water and consumed feed normally. At the end of the acclimatisation period, 30 mice were randomly divided into three groups according to body weight: normal control group (CON): fed normal chow (10% kcal from fat, XTCON50J, Xietong Pharmaceutical Bio-engineering Co., Jiangsu, China), high fat control group (HFD): fed high fat chow (60% kcal from fat, XTHF60, Xietong Pharmaceutical Bio-engineering Co., Jiangsu, China), and high fat-*L. plantarum* treatment group (HFD-ZW), with 10 mice in a group and every two in a cage (*n* = 5) (Table 1). The experimental period was 15 weeks, of which the obesity modelling lasted for 10 weeks with weekly weighing, but in week 11, mice in the HFD-ZW group received 150 μL of *Lactobacillus plantarum* HMRS-6 for 14 straight days, whereas those in the CON and HFD groups were administered an identical saline dose, and the mice were resumed the original food and water intake after 14 days until the last day of the experiments when the mice were put to death and the samples were collected for the subsequent analyses (Figure 1).

**Table 1:** Formula table of mice diet.

Ingredients	CON (kcal)	HFD (kcal)
Corn Starch	0.0	2024.8
Casein	800.0	800.0
Sucrose	291.2	291.2
Soybean oil	225.0	225.0
Lard	2205.0	180.0
L-cystine	12.0	12.0
Maltodextrin	500.0	500.0

### 2.2 Strain activation and oral gavage

*Lactobacillus plantarum* HMRS-6 was isolated from pickles in Hunan, China, and stored in 30% glycerol test tubes at −80°C in the Microbiology Laboratory, Hunan Agricultural University (Changsha, China). The bacteria were inoculated into the MRS activation medium (Solarbio Science & Technology Co., Ltd., Beijing, China) and placed in a shaker for 24 h at an incubation temperature of 25°C and a rotational speed of 180 rpm. 1 mL of activated bacterial solution was aspirated and diluted to obtain a gradient dilution of 10^−1^ to 10^−9^ bacterial solution, 100 mL of MRS solid medium was configured and distributed into multiple dry sterilised Petri dishes, and after solidification, 100 μL was aspirated after solidification, 100 μL of the gradient-diluted bacterial solution was added to the solid petri dishes and coated evenly, and the plates were counted after being inverted and incubated at 25°C for 24 h. The amount of bacterial solution for each mouse was 1 × 10^9^ CFU, divided by the number of colonies per milliliter counted on the plate, namely, the amount of bacterial solution for each mouse in the HFD-ZW group was 150 μL/d. The amount of bacterial solution required for each mouse per day was aspirated, and then placed into a centrifuge and centrifuged at 10,000 rpm for 15 min. After centrifugation, the supernatant was skimmed, and the bacterial solution was resuspended by adding an appropriate amount of sterile water to get the amount of bacterial solution for the daily gastric gavage.

### 2.3 Glucose and insulin tolerance tests

Mice underwent the Intraperitoneal Glucose Tolerance Test (IPGTT) following 10–14 h of fasting. Every mice received a gavage of 2.5 g/kg of glucose, followed by measuring their tail vein blood glucose levels using a glucometer for the subsequent 0, 15, 30, 60, and 120 min. Every mice received an intraperitoneal injection of 0.75 U/Kg insulin, followed by measuring their tail vein blood glucose levels using a glucometer at intervals of 0, 15, 30, 60, and 120 min. Mice underwent the Insulin Tolerance Test (ITT) following a fasting period of 10–14 h. Every mice received an intraperitoneal insulin injection, and their blood sugar levels in the tail vein were monitored using a glucometer at intervals of 0, 15, 30, 60, and 120 min.

### 2.4 Sampling and sample preservation

After completing the insulin resistance test and glucose tolerance test, blood was collected from mice into centrifuge tubes using the eyeball blood collection method. Following the gathering of blood, the mice underwent cervical dislocation, with rapid dissection, followed by the collection and weighing of subcutaneous and visceral fat. Subsequently, the mice colon samples were placed in centrifuge tubes and promptly immersed in liquid nitrogen for a brief period of rapid freezing. Post-sampling, the gathered blood underwent swift centrifugation at 3,500 rpm for a duration of 10 min in a freezing centrifuge. Subsequently, the clear liquid above the sediment was moved to a fresh centrifuge tube and chilled at 4°C, whereas the accumulated colon samples were kept in a refrigerator at −80°C.

### 2.5 Serum metabolome prep

The procedure involved naturally defrosting pre-stored mice serum, extracting 100 μL of this serum into a 1 mL centrifuge tube, then mixing it fourfold (400 μL) with a 1:1 methanol/acetonitrile solvent mixture. This mixture was agitated in a vortex shaker for 30 s, thoroughly mixed, ultrasonicated in an ice bath at 4°C for 10 min, refrigerated at −80°C for 8 h post-ultrasonication, and finally centrifuged for 15 min at 4°C and 12,000 rpm. Following a 15-min centrifugation, the clear liquid above the sediment was drawn into a fresh centrifuge tube and dehydrated to a dry state using a vacuum dryer. Introduce 100 μL of a solvent (methanol/acetonitrile), proceed to sonicate the mixture at 4°C for a duration of 10 min, followed by centrifugation at 4°C and 12,000 rpm for 15 min. Subsequently, transfer the supernatant into a fresh tube, and ultimately store the sample in an ultra-low temperature fridge at −80°C to freeze.

### 2.6 Ultrahigh-performance liquid chromatography-quadrupole time-of-flight mass spectrometry (UHPLC/Q-TOF-MS) analysis

Fifteen mice serum specimens were gathered in total, from which 10 μL was extracted, blended, and segmented into five parts, designated as a Quality control (QC) group to stabilize the device and adjust the data for pre-treatment. UHPLC: An Agilent 1290HPLC system; Column: Eclipse Plus C18, RRHD 1.8um, 2.1 × 100 mm, temperature set to 30°C; utilizing mobile phases: a mixture of 0.1% formic acid in water (Mobile phase A) and 0.1% formic acid acetonitrile (Mobile phase B). The flow rate was regulated to 0.3 mL/min, with a controlled injection volume of 2 μL, and the elution method is detailed in Table 2. The rate of ionization flow was established at 1.2 L/min, the interval for mass scanning ranged from 20 to 100 meters per second, and the temperature of the drying gas was fixed at 200°C. The data output from the mass spectrometer (Agilent 6,545 Q-TOF LC/MS) were processed and interpreted using the appropriate mass spectrometry software to determine the mass peak areas or peak heights of the target compounds and analyzed quantitatively by HPLC-MS/MS in combination with known standards.

**Table 2:** Elution procedure.

Time (min)	Mobile phase A (%)	Mobile phase B (%)	Flow velocity (mL/min)	Critical pressure (bar)
0	95	5	0.3	1,300
2	95	5	0.3	
20	0	100	0.3	
25	0	100	0.3	

### 2.7 Microbiota profiling by 16S rRNA amplicon sequencing

**T**otal mice intestinal microbial DNA was extracted from mouse colonic content species by referring to the QIAamp Power Fecal DNA kit instructions, and the V3V4 variable region of the mouse intestinal bacterial 16S rRNA gene was specifically amplified using the polymerase chain reaction (PCR), with the primers 27F 5′-AGRGTTTGATYNTGGCTCAG-3′ and 1492R 5′-TASGGHTACCTTGTTASGACTT-3′, in which each sample was repeated three times, the PCR products were purified by the kit, and then gel recovery was carried out by agarose gel electrophoresis at a concentration of 1.8%, and the results of the electrophoresis and PCR were imaged using a nucleic acid gel imager and characterised using ImageJ software. The recovered PCR products were subjected to bipartite sequencing and library construction using Illumina NovaSeq second-generation sequencing platform. Initial identification and quality control of the raw data was performed using QIIME2 (2023.6) software to remove low-quality, repetitive and short sequences (<100 bp) from the library. Sequencing splices during sequencing and library construction were removed using the Cutadapt program, sequence chimeras in the library were removed and sequencing fragments were spliced using the Usearch program, a filter condition of 97% similarity with a threshold of 0.005% was set, and OTU clustering and downscaling was performed on the filtered data, resulting in a total of 698 OTUs being identified for the complete study using the available faecal samples. QIIME2 was used to analyse the α-diversity and β-diversity of microorganisms in the samples, as well as the distribution of microorganisms in each treatment group at each taxonomic level and the dominant flora in each group.

### 2.8 Differential metabolite screening and pathway analysis

The raw data obtained were uploaded to Human Metabolome Database (HMDB) and MetaboAnalyst databases after noise reduction and data normalization using Metaboscape software, and the basic information of metabolites was obtained by preliminary retrieval. The screening conditions were VIP > 1, *p*-value<0.05, FC > 2. Finally, differential metabolite pathways were analyzed using the pathway analysis panel in the MetaboAnalyst website.^1^

### 2.9 Data and statistical analyses

The experiment’s data is presented as the mean plus or minus the standard error of the mean (SEM). The variance among group averages was examined through one-way ANOVA and evaluated via Duncan’s multiple comparison method. IBM SPSS Statistics 22 for Windows was utilized to conduct these studies. The correlation between colonic microorganisms and serum metabolites was assessed using Spearman’s correlation coefficient. Statistical significance was attributed to *P* < 0.05.

## 3 Results

### 3.1 *Lactobacillus plantarum* inhibits HFD-induced obesity in mice

**Figure 2:**
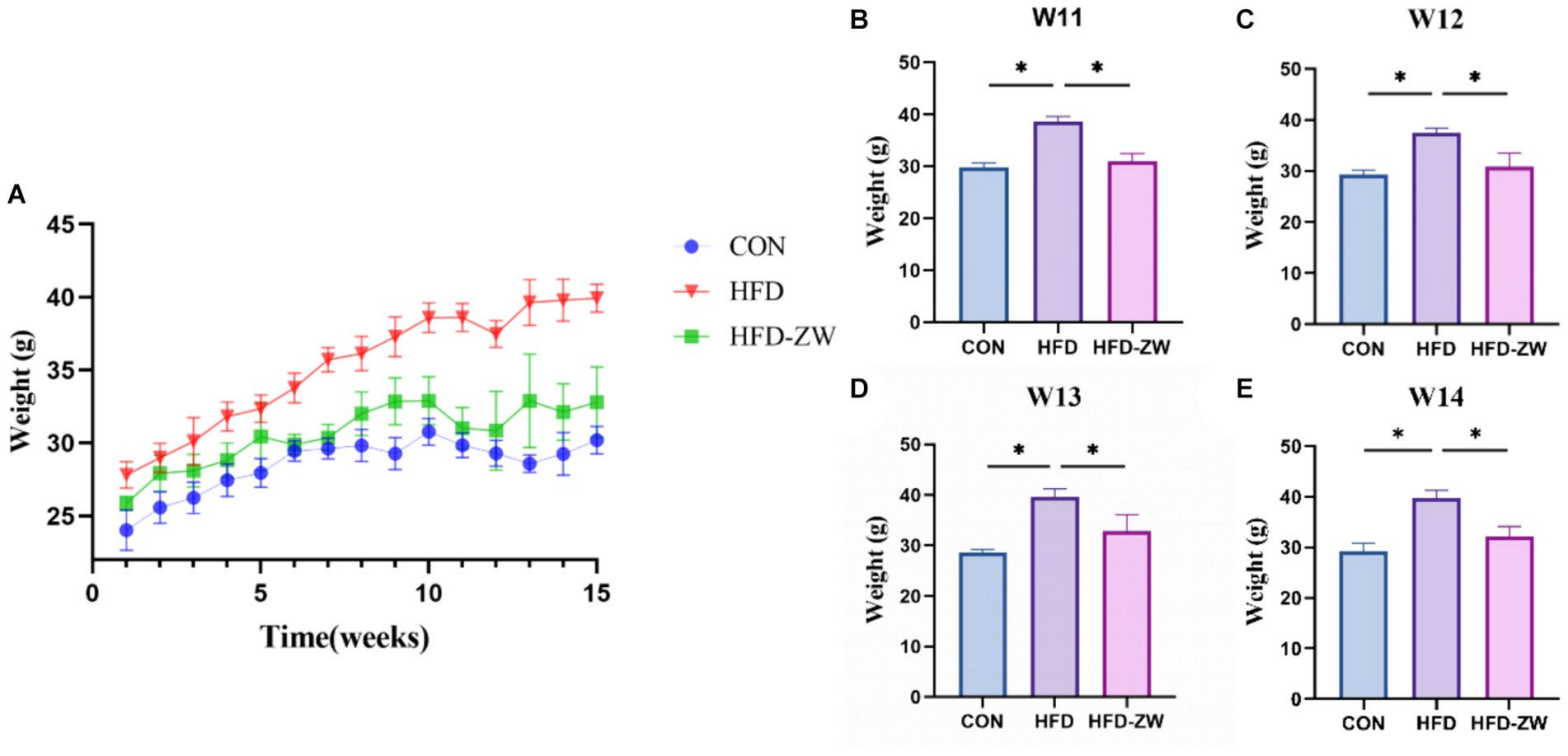
*L. plantarum* on body weight in obese mice. **(A)** Change in body weight of mice. **(B–E)** Weight changes in each treatment group during gavage. Data are means ± standard deviation and were analysed by one-way ANOVA. *p < 0.05 (*n* = 5).

From week 3, the body weights of mice in the high-fat-fed HFD and HFD-ZW groups were significantly higher than those in the regular-fed CON group (*p* < 0.05), and the most pronounced difference was observed at week 8 (Figure 2A), with the body weights of mice in the HFD group and the HFD-ZW group being approximately 1.21 and 1.07 times that of those in the CON group, respectively (*p* < 0.01). Prior to week 11, the average body weight of mice in the CON group was 30.55 g, whereas that of mice in the HFD and HFD-ZW groups was 38.97 g. The overweight rate of the HFD group was 27.56% compared with that of the standard CON group (*p* < 0.001). Starting from the 11th week, mice in the HFD-ZW group were intragastrically gavaged with 150 μL of *L. plantarum*, and both mice in the CON group and the HFD-ZW group showed different degrees of body weight loss in the first week of gavage, with a significant 5.72% decrease in body weight of the mice in the HFD-ZW group in the 11th week (Figure 2B). Meanwhile, significant differences in body weight were also observed between the HFD-ZW-treated group and the HFD modeling group (*p* < 0.05), and the gavage test showed that the body weights of each group decreased by 6.23 and 2.87%, respectively (Figures 2B–E), which indicated the therapeutic efficacy of *L. plantarum* administered by gavage on obesity and significantly reduced the body weights of the mice in the modeling group (*p* < 0.05).

### 3.2 *Lactobacillus plantarum* enhances glucose tolerance and insulin resistance in obese mice

**Figure 3:**
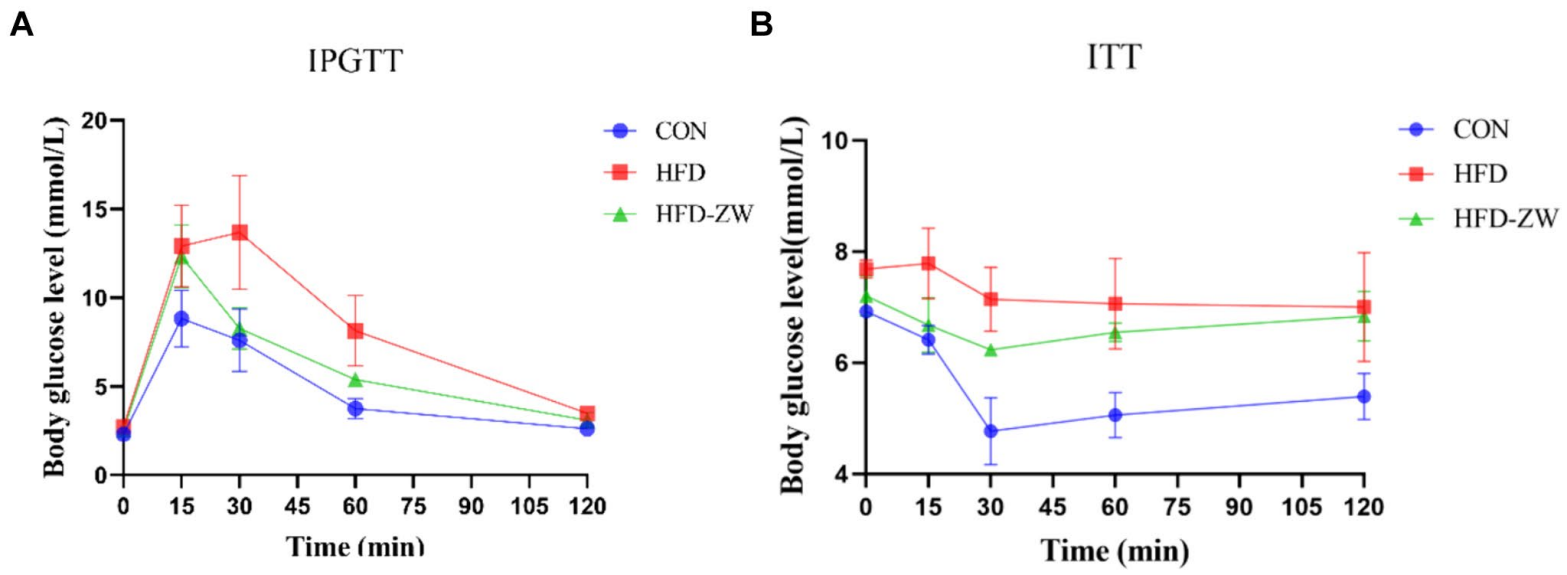
Effect of *L. plantarum* on glucose tolerance and insulin resistance in obese mice. **(A)** Plasma glucose levels in the mice glucose tolerance test; **(B)** plasma glucose levels in the mice insulin resistance test.

During the intraperitoneal glucose tolerance examination, it was observed that every mice exhibited a notable rise in blood sugar levels 15 min subsequent to receiving an intraperitoneal glucose injection (Figure 3A), yet in a span of 15–30 min, there was a notable reduction in the blood sugar levels of mice in the HFD-ZW group relative to those in the CON and HFD groups. Variations in blood sugar levels among mice during the glucose tolerance test consistently exhibited a pattern of rising and then falling, with the glucose area under the curve (AUC) in the HFD group mice notably exceeding that in the CON and HFD-ZW groups. The insulin resistance test revealed (Figure 3B). It was observed that the HFD group consistently exhibited the highest blood glucose levels, in contrast to the CON group, which had the lowest during the 0–120 min. The HFD group did not demonstrate a notable reduction in blood glucose levels in the 0–15 min period, in contrast to the other two groups, indicating a remarkable imbalance in insulin sensitivity.

### 3.3 *Lactobacillus plantarum* reduces HFD-induced adiposity gain and decreases adipocyte size

**Figure 4:**
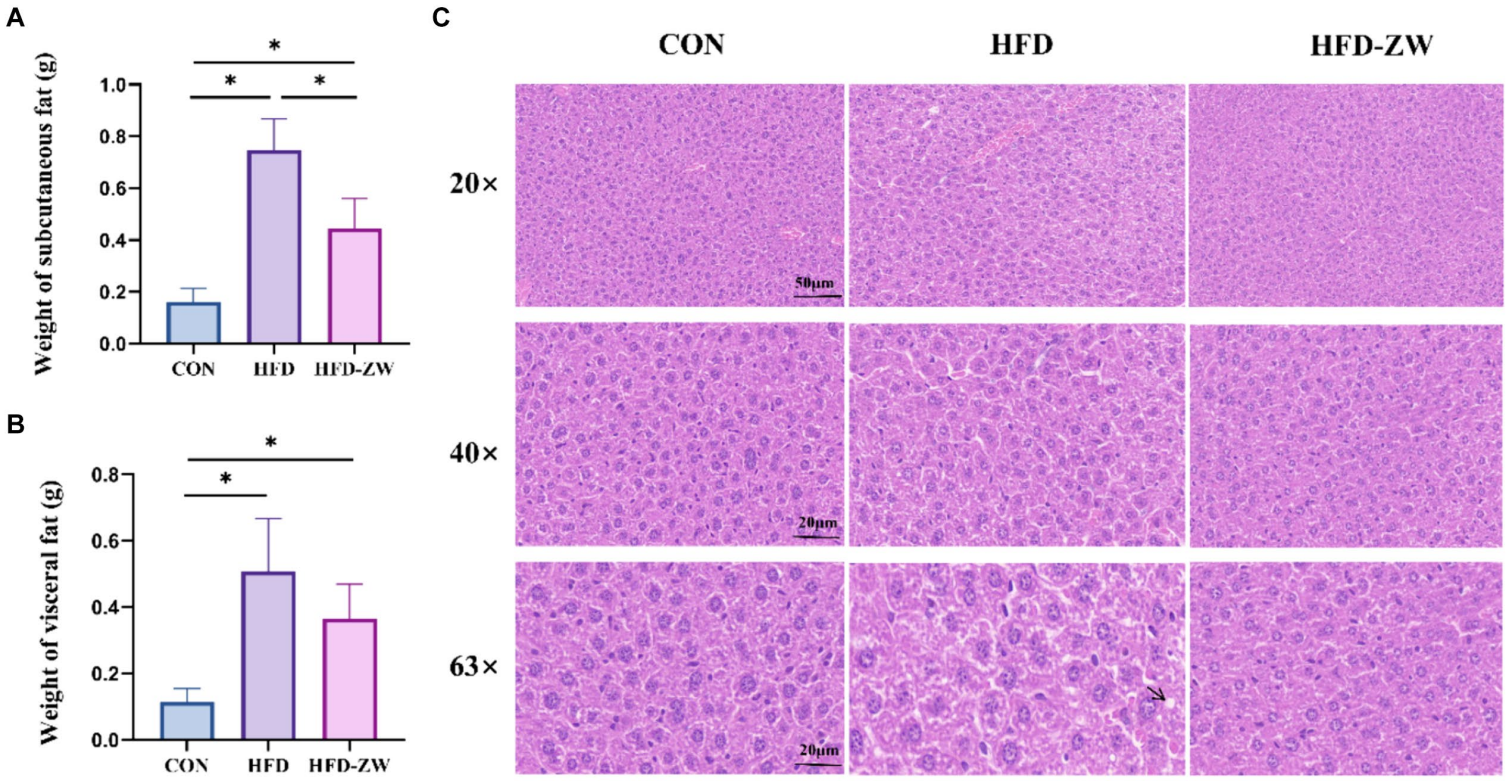
Effects of *L. plantarum* on adipocyte and liver histological changes in obese mice. **(A)** Changes in subcutaneous fat weight; **(B)** changes in visceral fat weight; **(C)** histological changes in liver sections measured by H&E staining with 20×, 40× and 63× magnification, respectively. Data are mean ± standard deviation and were analysed by one-way ANOVA. *p < 0.05 (*n* = 5).

The HFD group exhibited notably greater subcutaneous and visceral fat weights with regard to the CON group (*p* < 0.05). Additionally, the proportions of subcutaneous and visceral fat to body weight (%) were notably greater in the HFD group than in the CON and HFD-ZW groups (Figures 4A,B). Additionally, we conducted HE staining on both subcutaneous and visceral fat in mice, discovering a notable reduction in adipocyte size in the CON and HFD-ZW compared to the HFD group (*p* < 0.05). Consistently, accumulated lipid droplets and balloon-like structures, which are markers of hepatic steatosis, were observed in the hepatocytes of the HFD group (Figure 4C). After plant lactic rod treatment, lipid droplet size was significantly reduced and hepatic steatosis was significantly improved in the HFD-ZW in comparison to HFD-fed mice.

### 3.4 Analysis of microbial diversity in the mice colon

**Figure 5:**
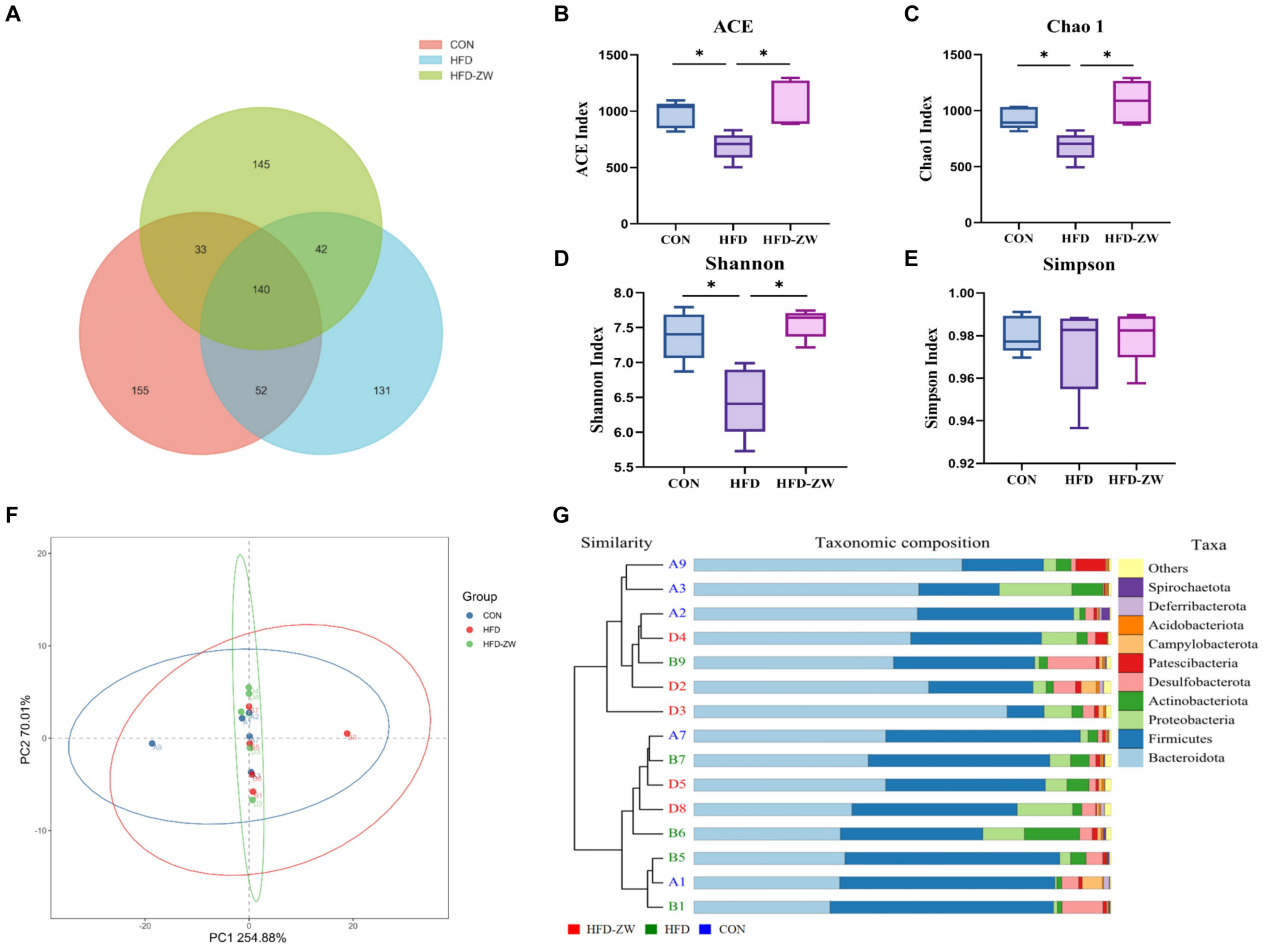
Effect of *L. plantarum* treatment on microbial species richness, α and β diversity indices in mice colon. **(A)** Venn diagram of OTU distribution. **(B)** ACE index. **(C)** Chao 1 index. **(D)** Shannon’s index. **(E)** Simpson’s index. **(F)** Principal co-ordinate analysis (PCoA) showing microbial community distances between baseline of the CON group (blue circles), the HFD group (red circles), and the baseline of the group after *L. plantarum* intervention (green circles). **(G)** Unweighted pair group method with arithmetic mean (UPGMA) analysis. Data are means ± standard deviation and were analyzed using one-way ANOVA. *p < 0.05 (*n* = 5).

The distribution of OTUs in each group was as follows: 380 in the CON group, 365 in the HFD group, and 360 in the HFD-ZW group, of which the number of featured articles was 155, 135, and 145, respectively (Figure 5A). From this, it can be preliminarily hypothesised that obesity had reduced the species richness of the microorganisms of the mice colon to a greater extent, and that *L. plantarum* gastric gavage had restored the species richness of the microorganisms of the mice intestine to a certain degree.

According to the analysis of α-diversity indices of each treatment group, ACE and Chao1 indices could be used to measure the number of species (Figures 5B,C), and Shannon and Simpson indices were used to assess the diversity of species (Figures 5D,E). The ACE, Shannon and Chao1 indices of the HFD group had the lowest values among all treatment groups, and the three indices were significantly higher in the HFD-ZW and CON groups (*p* < 0.05), and the ACE, Shannon and Chao1 indices tended to increase in the HFD-ZW with regard to the CON group, which indicated that *L. plantarum* gavage increased the species richness of mice colon microorganisms to a greater extent, which was consistent with the results of the OTU distribution; the α-diversity indices of the HFD group were lower than those of the other treatment groups, which may indicate that the obesity environment reduced the microbial diversity of the mice colon to a greater extent. In addition to measure the degree of similarity between microbial communities, β-diversity was further assessed using Binary-jaccard PCoA. In this study, PCoA was used to analyse the microbial community structure of colon contents from different groups of mice. The results showed that the probiotic group had the greatest variability after the intervention *L. plantarum* after the intervention (Figure 5F). However, the UPGMA results showed that the HFD-ZW group was closer to the CON group than the HFD group at the phylum level (Figure 5G).

### 3.5 Analysis of colonic microflora composition in mice

#### 3.5.1 *Lactobacillus plantarum* affects the abundance of microorganisms at phylum level under high fat diet induced obesity

**Figure 6:**
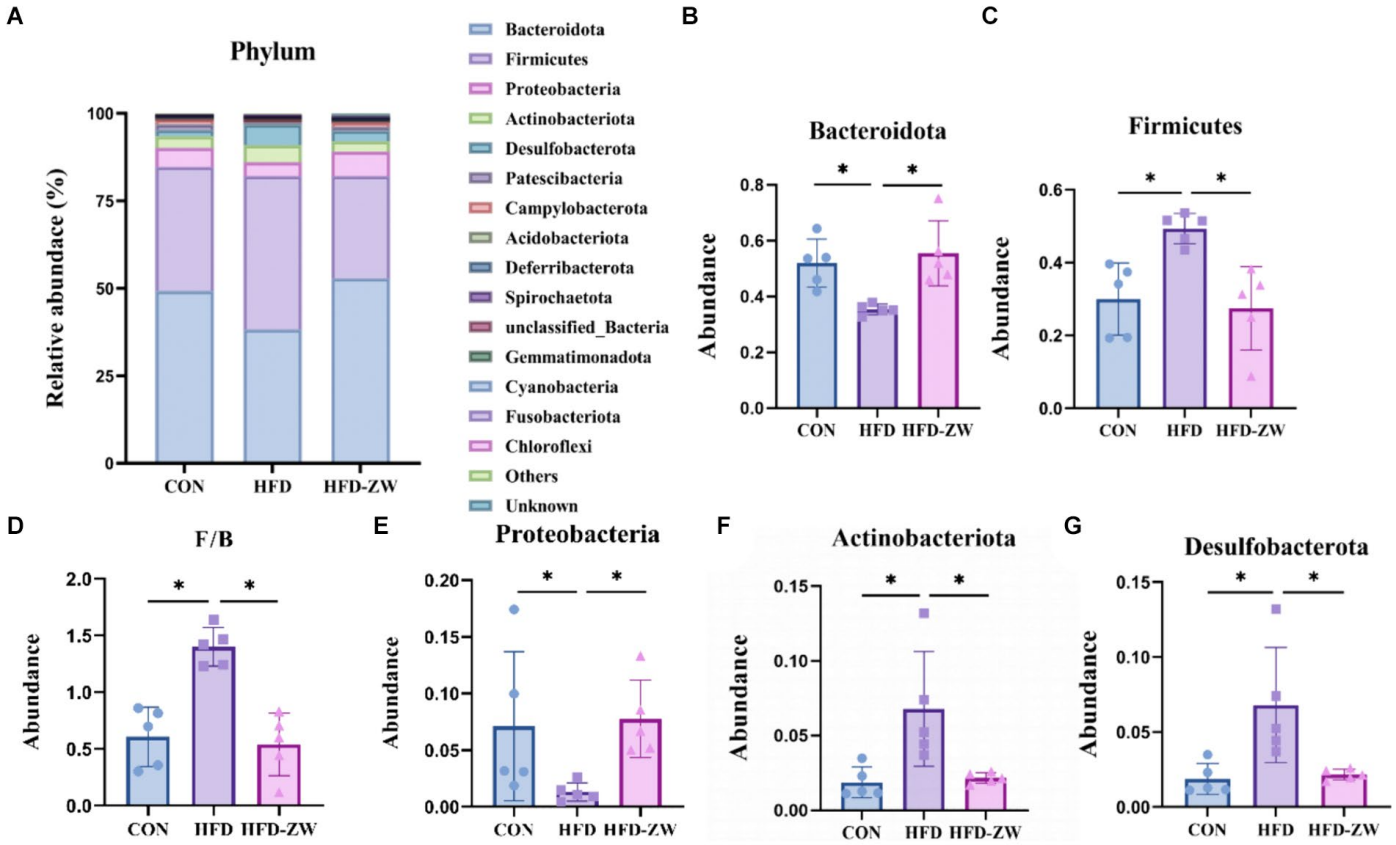
Analysis of microbial components at the phylum level. **(A)** Relative abundance of microbial phyla in the mice colon. Components of the relative abundance of the Bacteoidota **(B)**, Firmicutes **(C)**, F/B ratio **(D)**, Proteobacteria **(E)**, Actinobacteria **(F)**, and Desulfobacterota **(G)** in the colons of mice in the CON, HFD, and HFD-ZW groups. *p < 0.05 (*n* = 5).

Within the phylum, the leading five microbial phyla were Bacteroidota, Firmicutes, Proteobacteria, Actinobacteriota, and Desulfobacterota, representing over 90% of the overall count (Figure 6A). In each group, the relative abundance of Bacteroidetes was 49.32, 38.31, and 52.90% (Figure 6B), and the high-fat environment significantly decreased the abundance of Bacteroidetes, on the contrary, the gavage of *L. plantarum* significantly increased the relative abundance of Bacteroidetes in the colons of the mice (*p* < 0.05), and even marginally exceeded the CON group’s by 3.58%. Meanwhile, as the relative abundance of the Firmicutes in the HFD group increased, its relative abundance in the CON and HFD-ZW groups was 35.40 and 29.19%, respectively (Figure 6C), 8.29 and 14.6%, respectively, lower than in the HFD group. Consequently, there was a notable rise in the proportion of F/B within the HFD group (Figure 6D). Furthermore, the comparative prevalence of the Proteobacteria in each experimental group stood at 5.47, 3.95, and 7.06% correspondingly (Figure 6E), indicating a downward trend in the HFD group, yet the difference was not statistically meaningful. Both Actinobacteria phylum and Desulfobacterota significantly reversed the increasing trend in high-fat environment after gavage by *L. plantarum* (Figures 6F,G) (*p* < 0.05).

#### 3.5.2 *Lactobacillus plantarum* affects the abundance of microorganisms at class level under high fat diet induced obesity

**Figure 7:**
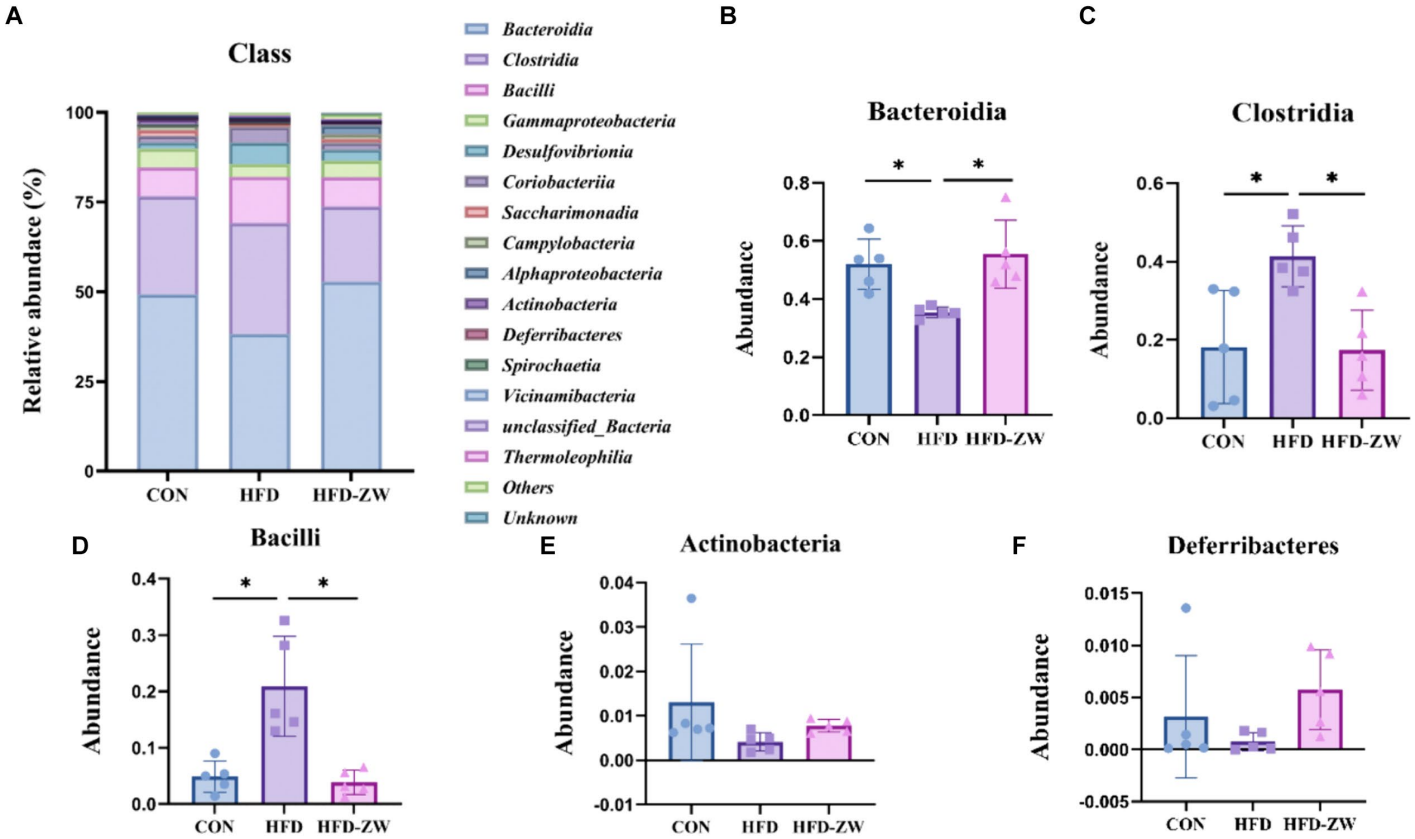
Analysis of microbial components at the class level. **(A)** Relative abundance of microbial class in the mice colon. Components of the relative abundance of Bacteroidia **(B)**, Clostridia **(C)**, Bacilli **(D)**, Actinobacteria **(E)**, and Deferribacteres **(F)** in the colons of mice in the CON, HFD, and HFD-ZW groups. *p < 0.05 (*n* = 5).

The microbial composition in the mice colon was examined, revealing the top 15 microorganisms at the Class level, as illustrated in Figure 7A. Notably, the predominant microorganisms, including Bacteroidia, Clostridia, Bacilli, Actinobacteria, and Deferribacteres, collectively accounted for more than 80% of the total microbial population. Specifically, under high-fat conditions, a reduction of 11.01% in the relative abundance of Bacteroidia was observed (Figure 7B), mirroring the trends seen in the Bacteroidota phylum. However, administration of *L. plantarum* through gavage resulted in a significant increase in the relative abundance of Bacteroidia (*p* < 0.05). In the CON, HFD, and HFD-ZW groups, the relative abundance of Clostridia was found to be 27.35, 30.90, and 20.90%, respectively (Figure 7C). Similarly, the relative abundance of Bacilli was observed to be 7.98, 12.82, and 8.20%, respectively (Figure 7D). Notably, both the CON group and the HFD-ZW group exhibited a significant increasing trend in Bacilli abundance. Additionally, the relative abundance of Actinobacteria was found to be 1.81, 5.94, and 3.03% (Figure 7E), while the relative abundance of Deferribacteres in each treatment group was 5.29, 3.69, and 4.68%, with no statistically significant difference observed (*p* > 0.05) (Figure 7F). Comparative analysis revealed a significant increase in the abundance of Clostridia and Bacilli (*p* < 0.05) in the HFD group compared to CON. Importantly, administration of *L. plantarum* effectively restored the altered microbial abundance induced by the high-fat conditions.

#### 3.5.3 *Lactobacillus plantarum* affects microbial abundance at the genus level under high-fat diet-induced obesity

**Figure 8:**
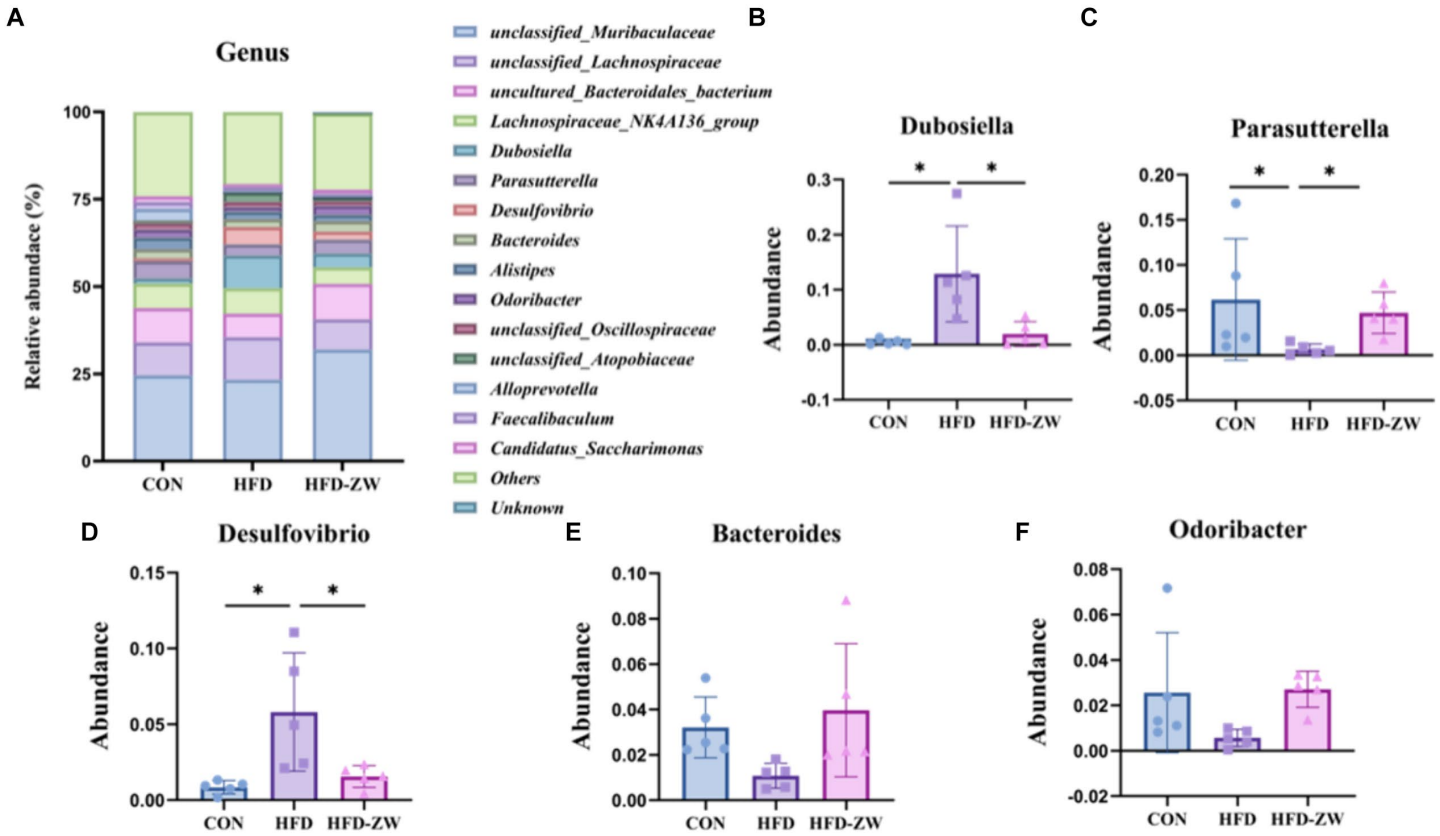
Analysis of microbial components at the genus level. **(A)** Relative abundance of microbial genus in the mice colon. Components of the relative abundance of *Dubosiella* **(B)**, *Parasutterella* **(C)**, *Desulfovibrio* **(D)**, *Bacteroides* **(E)**, and *Odoribacter* **(F)** in the colons of mice in the CON, HFD, and HFD-ZW groups. *p < 0.05 (*n* = 5).

By analysing the composition of microorganisms in the mice colon, the five dominant genera at the level of the 15 most abundant genera were *Dubosiella*, *Parasutterella*, *Desulfobibrio*, *Bacteroides* and *Odoribacter* (Figure 8A). *Parasutterella*, *Bacteroides*, and *Odoribacter* decreased by 1.66, 0.37, and 0.97%, respectively, under high-fat conditions compared to the CON group (Figures 8C,E,F). In addition, the relative abundance of *Dubosiella* was 1.54, 9.37, and 4.01% (Figure 8B), and that of *Desulfobibrio* was 0.83, 4.99, and 2.27% (Figure 8D), respectively, which were significantly increased (*p* < 0.05) in the HFD group. Whereas, using *L. plantarum* gavage treatment, *Parasutterella*, *Bacteroides* and *Odoribacter* all had a tendency to recover the same abundance as that of the CON group (Figures 8C,E,F), but there was no statistical difference. Meanwhile, *Dubosiella* and *Desulfobibrio* in the HFD-ZW group reversed the abundance changes induced by the high-fat environment (Figures 8B,D) and showed a significant recovery in abundance (*p* < 0.05).

### 3.6 LEfSe analysis of microbiota in colon contents

**Figure 9:**
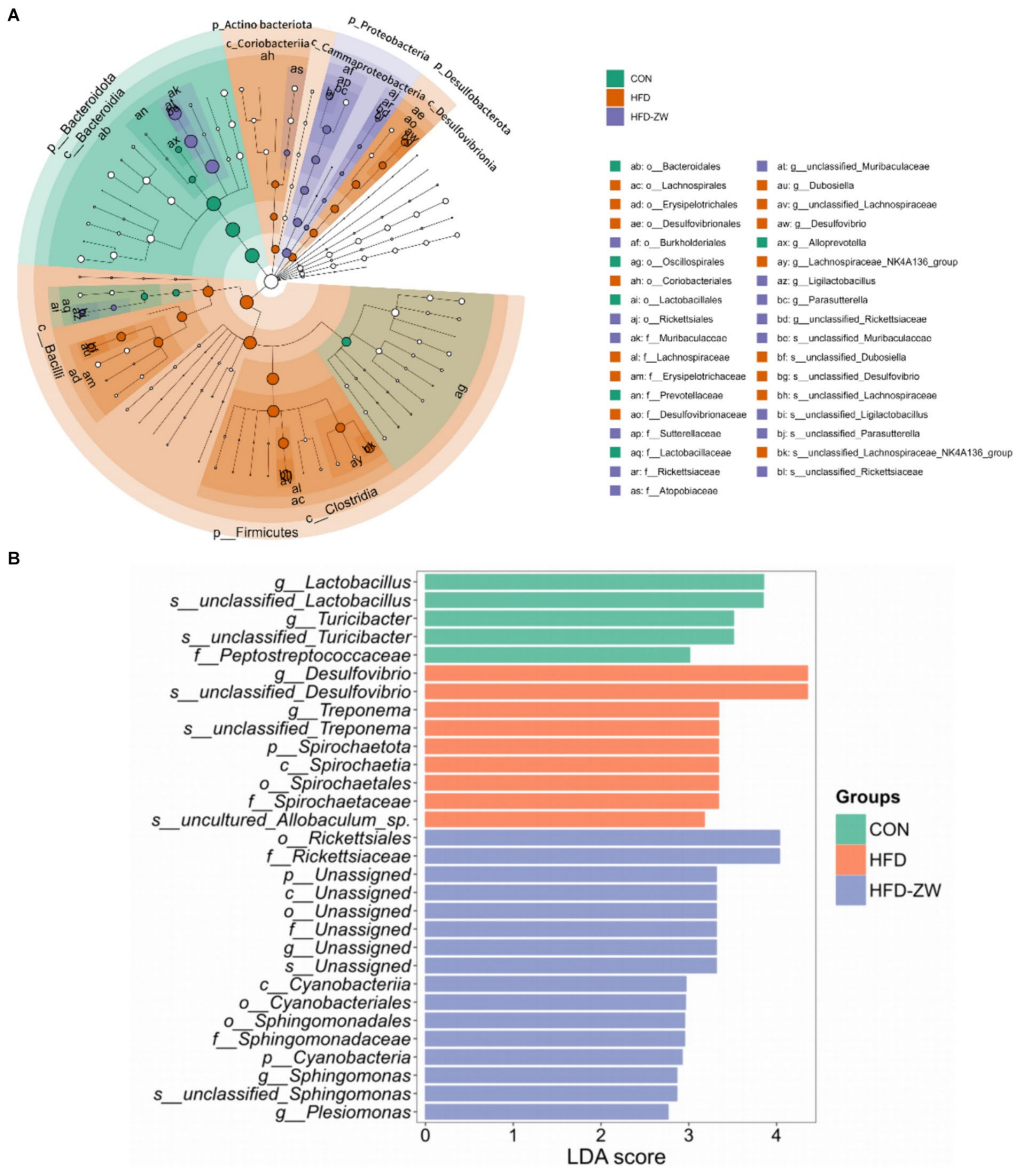
Characterisation of mice colonic microorganisms identified by *L. plantarum* treatment. **(A)** LEfSe taxonomic branching diagram, different colours indicate enrichment of certain taxonomic units in the CON group (green), the HFD group (orange) and the HFD-ZW group (purple); **(B)** LDA scores, LDA scores higher than 2 were considered to be significant contributors to the model.

The results of LEfSe analysis of colonic microorganisms in the CON, HFD, and HFD-ZW groups are shown in Figure 9A (*p* < 0.05). We found that the order of significant enrichment in the CON group was Bacteroidales, Oscillospirales, and Lacteobacillales; the families Prevotellaceae and Lactobacillaceae; and the genus *Alloprevotella*. The HFD group was remarkably enriched in the genera Lachnospirales, Erysipelotrichales, Desulfovibrionales, order Coriobacteriales; families Lachnospiraceae, Erysipelotrichaceae, Desulfovibrionaceae; *Dubosiella*, *unclassified_Lachnospireaceae*, *Desulfovibrio* and *Lachnospiraceae_NK4A136_groups* were significantly enriched. The HFD-ZW group was in the orders Burkholderiales, Richettsiales; Families Muribaculaceae, Sutterellaceae, Rickettsiaceae, Atopobiaceae; *unclassified_ Muribaculaceae*, *Ligilactobacillus*, *Parasutterella*, *unclassified_ Rickettsiaceae* genera were significantly enriched. By identifying Linear Discriminant Analysis (LDA) scores above 2 (Figure 9B), it was possible to determine that species abundance differed in each group. In the HFD-ZW group, the order Cyanobacteriia; the orders Richettsiales, Cyanobacteriia, Cyannobacteriales, Sphingomonadales; the families Rickettsiaceae, Sphingomonadaceae; The species abundance of the genera *Spingomonas*, *unclassified_Sphingomonas*, and *Plesiomonas* had the most notably effect on the abundance of differential bacteria. Further revealed significant differences in biological branching composition between the control, HFD and HFD-ZW groups, which is consistent with the above results.

### 3.7 Influence of *Lactobacillus plantarum* treatment on serum levels of total metabolites in mice

**Figure 10:**
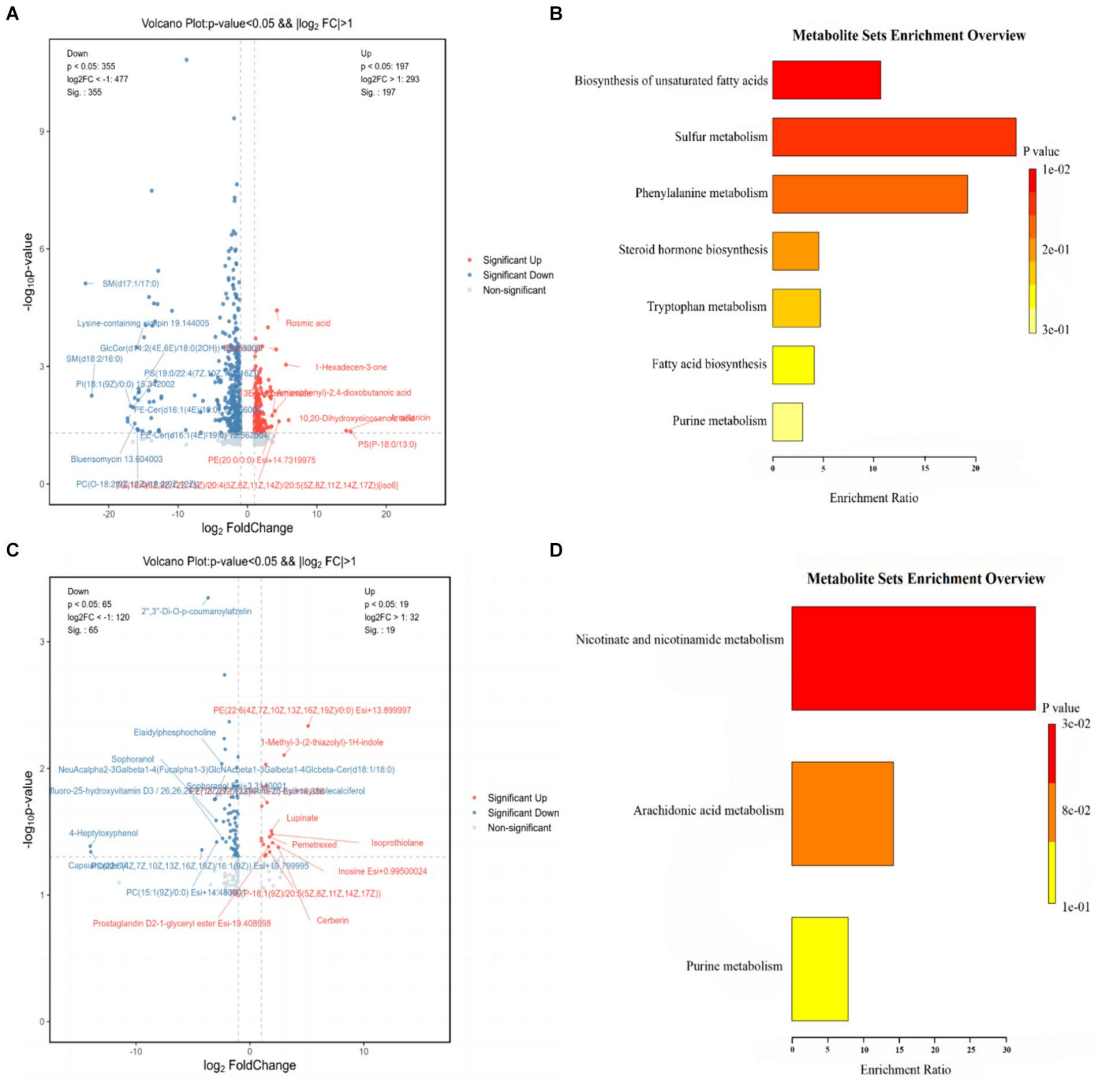
Differential metabolite and KEGG pathway enrichment analysis results. **(A)** Analysis of differential metabolites between CON and HFD; **(B)** results of enrichment analysis of KEGG pathways between CON and HFD; **(C)** analysis of differential metabolites between HFD and HFD-ZW; **(D)** results of enrichment analysis of KEGG pathways between HFD and HFD-ZW.

The serum differential metabolites of mice from the CON, HFD, and HFD-ZW groups were comprehensively analyzed through pairwise comparisons, as depicted in Figure 10. Notably, metabolites with *p*-values <0.05 and FC-values >1 were considered significant. The differential metabolite volcano plot revealed a total of 554 distinctive metabolites between the CON and HFD groups (Figure 10A). Among these, Rosmic acid, Edulisin III, 1-Hexadecen-3-one, Armillaricin, PS (18:0/13:0), PE (20:0/0:0), and 10,20-Dihydroxyeicosanoic acid were found to be significantly up-regulated in the CON group. On the other hand, there were 84 differential metabolites identified between the HFD and HFD-ZW groups (Figure 10C). Following the administration of *Lactobacillus plantarum* gavage treatment, metabolites including PE (22:6(4Z,7Z,10Z,13Z,16Z,19Z)/0:0), 1-Methyl-3-(2-thiazolyl)-1H-indole, Lupinate, Pemetrexed, Isoprothiolane, Inosine, and Cerberin showed significant up-regulation, whereas Sophoranol, 4-Heptyloxyphenol, and Capsianoside VI displayed decreased expression. Notably, similar trends were observed in the CON group for metabolites such as PE-Cer(d16:1(4E)/19:0), Cycloartanyl ferulate, Cer(d18:1/24:1), and Tetrahydroaldosterone-3-glucuronide following *L. plantarum* treatment.

The results of the Kyoto Encyclopedia of Genes and Genomes (KEGG) database enrichment analysis of the major differentiated metabolites indicated distinct pathways associated with unsaturated fatty acid biosynthesis, sulfur metabolism, phenylalanine metabolism, steroid hormone biosynthesis, tryptophan metabolism, fatty acid biosynthesis, and purine metabolism between the CON and the HFD group (Figure 10B). In contrast, differential metabolites in the HFD and HFD-ZW groups were largely enriched in domains of nicotinic acid and nicotinamide metabolism, arachidonic acid metabolism, and purine metabolism (Figure 10D). These findings provide valuable insights into the metabolic alterations associated with high-fat diet-induced conditions, as well as the potential modulatory effects of *L. plantarum* intervention.

### 3.8 Correlation between intestinal microbes and serum metabolites

**Figure 11:**
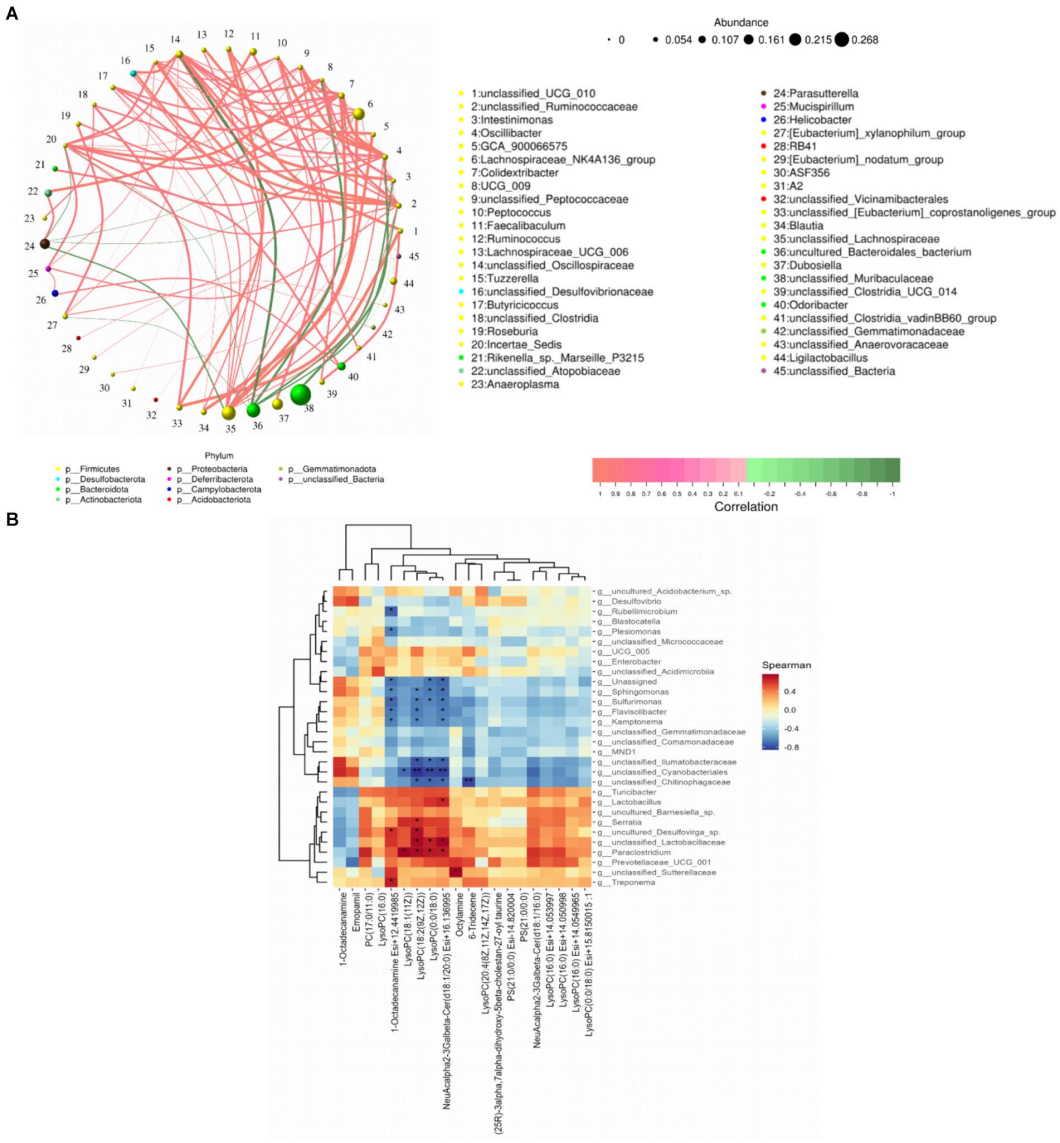
Spearman correlation analysis of gut flora with serum metabolites. **(A)** Network plot of gut flora correlation in mice; **(B)** heat map of gut differential microbiota versus raw serum differential metabolites. *p < 0.05 and **p < 0.01.

The genus-level network Spearman correlation analysis of the intestinal flora of each group of mice (Figure 11A) revealed that unclassified_UCG_010, unclassified_Ruminococcaceae, Colidextribacter, and Lachnospiraceae_UCG_006 showed high correlation with other microorganisms showed high positive correlation, while unclassified_Oscillospiraceae, Parasutterella, unclassified_Ruminococcaceae and Incertae_Sedis presented high negative correlation. And the relative abundance of Oscillibacter, unclassified_Ruminococcaceae, Incertae_Sedis and Lachnospiraceae_NK4A136_group was found to be at a high level according to node size. By analysing the heatmap of mice colon microbial correlation with serum differential metabolites it was found (Figure 11B) that LysoPC (18:1 (11Z)) showed a significant positive correlation with Paraclostridium, unclassified_Lactobacillaceae, uncultured_Barnesiella_sp. and Serratia were significantly positively correlated with each other. While unclassified_Cyanobacteriales and Sulfurimonas were significantly negatively correlated with LysoPC (18:2 (9Z, 12Z)), LysoPC (0:0/18:0).

## 4 Conclusion

This research delved into the healing capabilities of *L. plantarum*, a probiotic known for its diverse bioactivities (Li et al., 2020), by inducing obesity in mice on a high-fat diet and tube-feeding them with a probiotic with a wide range of bioactivities. The study showed that *L. plantarum* significantly alleviated and reduced body weight in obese mice. Besides, it can inhibit the accumulation of lipids in the liver. *L. plantarum* treatment promoted the restoration of glucose tolerance and insulin sensitivity in obese mice according to IPGTT and ITT results. Analysis of mice colon samples revealed that treatment with *L. plantarum* enhanced the alpha variety in intestinal microbes. There was a rise in the number of advantageous bacteria, a fall in the quantity of detrimental bacteria, and a reduction in the balance between Firmicutes and Bacteroidota. Serum metabolomics analysis revealed notable alterations in serum metabolites across all three mice groups. During obesity modelling and *L. plantarum* treatment, *L. plantarum* significantly increased Isoprothiolane, Inosine and Cerberin levels, inhibited lipid accumulation in adipocytes as well as facilitated fat burning, leading to obesity alleviation. Meanwhile the differential metabolites were significantly enriched in purine metabolism. These findings suggest that *L. plantarum* is expected to be able to be a new strategy for the effective treatment of obesity, providing a more optimised alternative to achieve therapeutic goals. In addition, more future studies are needed to explore in depth and comprehensively the specific mechanisms involved in alleviating obesity and to clarify the dosage of *L. plantarum* treatment. This will help to maximise therapeutic benefits when managing obesity.

## 5 Discussion

Obesity and its associated chronic diseases have become an important risk factor threatening global health (O'Neill and O'Driscoll, 2015). Several studies have proposed the potential efficacy of probiotics in the effective alleviation and treatment of obesity due to their modulation of gut microbial composition and maintenance of the functional integrity of the intestinal barrier (In Kim et al., 2019; Quigley, 2019). Thus, *L. plantarum*, which has a variety of bioactive functions, was selected for our study to investigate its efficacy in ameliorating obesity (Zhu et al., 2019; Park and Oh, 2007). It was found that treatment of high-fat diet-induced obesity by *L. plantarum* reduced body weight, subcutaneous and visceral fat weight as well as reduced lipid droplet size in the liver and improved liver function in obese mice (Leung et al., 2016; Wei et al., 2023). The data suggest that *L. plantarum* improves glucose tolerance, insulin resistance and regulates serum metabolites (Han et al., 2020). It is consistent with the findings of Ho et al. on the anti-obesity potential of *Lactobacillus rhamnosus* SG069 (LR069) and *Lactobacillus brevis* SG031 (LB031) as well as their ability in preventing diet-induced insulin resistance (Ho et al., 2024).

There exists a tight association between blood glucose and lipid metabolism (Kojta et al., 2020). Studies have shown that when the blood glucose level in the host body exceeds a certain threshold, the acetyl CoA, citric acid and ultimately ATP generated during the oxidative decomposition of glucose, the three can be allosterically activated acetyl CoA carboxylase, which ultimately leads to the conversion of glucose into fat stored in the adipose tissue (Saltiel and Kahn, 2001). However, we found that obese mice treated with *L. plantarum* by gavage had the ability to regulate blood glucose levels compared with the model group, as shown by the significant reduction of blood glucose levels in the treated group of obese mice 30 min after receiving intraperitoneal glucose injection. This indicates that *L. plantarum* is able to ameliorate the abnormal elevation of blood glucose values and reduce the conversion of glycogen into energy storage in the form of fat (Youn et al., 2021). Meanwhile, insulin plays a major role in reducing plasma glucose levels by controlling hepatic glucose metabolism. It was found by insulin resistance experiments that mice fed under high-fat conditions consistently had the highest blood glucose levels and within 15 min for showed the ability to lower blood glucose. However, both the HFD-ZW group and the CON group showed a tendency to lower blood glucose at 15 min, it is consistent with the findings of Zhao et al. (2022) and further indicates that *Lactobacillus plantarum* tube-feeding-treated mice were able to inhibit the level of insulin resistance in high-fat mice to a certain extent (Paranjape et al., 2010; Chao et al., 2019).

Notable alterations (*p* < 0.05) in the gut microbe makeup of mice were noted during the development of the obesity model and the ensuing application of *L. plantarum* (Moorthy et al., 2021). A notable reduction in Bacteroidota was noted in obese mice nurtured in a high-fat setting, contrasted by a marked rise in the HFD-ZW group (Furet et al., 2010). At the same time, the abundance of Firmicutes increased in the HFD group (Rastmanesh, 2011), which led to an increasing trend in the ratio of Firmicutes to Bacteroidota in high-fat conditions. It is well known that the elevated F/B ratio is widely recognized as a key factor in the emergence and advancement of obesity (Chang et al., 2015). It was found that Firmicutes can produce more harvestable energy than Bacteroidota, so that in the presence of an increase in the relative abundance of Firmicutes and a decrease in the relative abundance of Bacteroidota, calorie uptake is increased, which then promotes obesity (Ma et al., 2022; Li et al., 2020). Subsequently, HFD-fed obese mice significantly restored the F/B ratio to normal levels after treatment with *L. plantarum* gavage (Stojanov et al., 2020). This conclusion was also confirmed by a study on *Lactobacillus gasseri* (de Moura et al., 2023). In addition, we found that *L. plantarum* dietary intervention also reduced *Desulfovibrio* expression in the HFD group (Wang et al., 2020). It has been proposed that increased *Desulfovibrio* abundance is a key feature of obesity (Katamoto et al., 1991). At the same time with the increase of *Desulfovibrio* there is an antagonistic effect on Clostridia. High abundance of *Desulfovibrio* and low abundance of Clostridia ultimately lead to a decrease in the expression of CD36 (Buttet et al., 2014), a key regulator of lipid absorption in the intestinal tract, and its deficiency leads to lipid metabolism disorders and excessive accumulation of adipocytes in the body (Buttet et al., 2016), which in turn increases the chances of obesity and metabolic syndrome caused by HFD feeding (Ma et al., 2022). More notably, we found that tube-feeding treatment with *L. plantarum* also reduced the expression of *Clostridium difficile* in the HFD group. A previous study by Ma Yong et al. also found that high abundance of Clostridium can metabolise and produce excess faecal deoxycholic acid (DCA). This ecological disturbance further reduced bile acid uncoupling, and DCA stimulated S1PR2 expression ultimately inducing activation of NLRP3 inflammatory vesicles (Ma et al., 2022). Furthermore, *Dubosiella* abundance was significantly reduced following *L. plantarum* gavage treatment, showing a positive association with weight increase and a reduction in the proliferation of short-chain fatty acid (SCFA) producing bacteria (Qiu et al., 2021; Martin-Gallausiaux et al., 2021). Mariana et al. study similarly found that disturbances in the intestinal flora were negatively correlated with total SCFA production and were able to reverse this adverse effect after probiotic intervention (de Moura et al., 2023). These bacteria are synthesized by intestinal Bacteroides through the fermentation of polysaccharides and carbohydrates, which pose challenges for the host to assimilate and digest (Blaak et al., 2020). Also, *L. plantarum* was able to enhance the expression of Bacteroides, and these results suggest that it has a role in alleviating obesity (Backhed et al., 2004; Parnell and Reimer, 2012).

By analysing serum metabolomics, it was shown that metabolites PS-18:0/13:0 and PE (20:0/0:0) were significantly up-regulated in HFD-fed obese mice, PS-18:0/13:0 and PE (20:0/0:0) are important phospholipids in cell membranes and one of the important components of lipid metabolism (Xie et al., 2023), so it can be hypothesized that obese environments significantly reduce the metabolism of mice role in lipid metabolism in mice (Martinic et al., 2018). Serum metabolomics research was employed to evaluate the effectiveness of treating obese mice with *L. plantarum* and to understand its fundamental mechanisms. In obese mice treated with *L. plantarum*, the varied metabolites observed were PE (22:6(4Z,7Z,10Z,13Z,16Z,19Z)/0:0), 1-Methyl-3-(2-thiazolyl)-1H-indole, Pemetrexed, Isoprothiolane, Inosine, Cerberin, Cer(d18:1/24:1), 4-Heptyloxyphenol and Capsianoside VI. There was a notable rise in Isoprothiolane concentrations in the HFD-ZW relative to the HFD group. The study demonstrated that Isoprothiolane increased the levels of non-esterified fatty acids (NEFA) and was able to block lipid deposition into adipocytes (Katamoto et al., 1991), while having a common effect by accelerating the desaturation of fatty acids in tissue lipids. It is well known that obesity occurs as a result of excessive energy intake accumulating in the body in the form of fat, and increased Inosine expression promotes fat burning (Crunkhorn, 2022). Cer(d18:1/24:1) resumed the same decreasing trend as the CON group after *L. plantarum* gavage treatment (Yu et al., 2023). This is consistent with the findings of Yu Baowen et al. It was proposed that Cer(d18:1/24:1) levels are increased in the metabolic profile of obese subjects, which leads to reduced insulin sensitivity and is associated with metabolic disturbances in obesity and may also contribute to obesity comorbidities (Yu et al., 2023). The study suggests that increased levels of Cer(d18:1/24:1) may be a potential biomarker for obesity (Yu et al., 2023). Analysis of differential metabolites for potential signalling pathways involved in obesity showed that the CON vs. HFD group and the HFD vs. HFD-ZW group of varied microorganisms showed a notable increase in purine metabolism.

## Data Availability

The datasets presented in this study can be found in online repositories. The names of the repository/repositories and accession number(s) can be found at: https://www.ncbi.nlm.nih.gov/genbank/, PRJNA1104806.

## Footnotes

^1^https://genap.metaboanalyst.ca/MetaboAnalyst/home.xhtml
